# Medicinal Mushrooms: Bioactive Compounds, Use, and Clinical Trials

**DOI:** 10.3390/ijms22020634

**Published:** 2021-01-10

**Authors:** Giuseppe Venturella, Valeria Ferraro, Fortunato Cirlincione, Maria Letizia Gargano

**Affiliations:** 1 Department of Agricultural, Food and Forest Sciences, University of Palermo, Viale delle Scienze, Bldg. 5, I-90128 Palermo, Italy; valeria.ferraro@unipa.it (V.F.); fortunato.cirlincione@unipa.it (F.C.); 2 Department of Agricultural and Environmental Science, University of Bari Aldo Moro, Via Amendola 165/A, I-70126 Bari, Italy; marialetizia.gargano@uniba.it

**Keywords:** medicinal mushrooms, pharmaceutical properties, biomolecules, immunomodulation, antitumor property, dietary supplements, in vitro study, clinical trial, mycotherapy

## Abstract

Medicinal mushrooms have important health benefits and exhibit a broad spectrum of pharmacological activities, including antiallergic, antibacterial, antifungal, anti-inflammatory, antioxidative, antiviral, cytotoxic, immunomodulating, antidepressive, antihyperlipidemic, antidiabetic, digestive, hepatoprotective, neuroprotective, nephroprotective, osteoprotective, and hypotensive activities. The growing interest in mycotherapy requires a strong commitment from the scientific community to expand clinical trials and to propose supplements of safe origin and genetic purity. Bioactive compounds of selected medicinal mushrooms and their effects and mechanisms in in vitro and in vivo clinical studies are reported in this review. Besides, we analyzed the therapeutic use and pharmacological activities of mushrooms.

## 1. Introduction

Mushrooms, which have always been appreciated for their culinary and nutritional value, are now increasingly valued for their many important medicinal properties, so much so that they are used not only as dietary food (functional foods) but also in the form of dietary supplements, nutraceuticals, and mycotherapy products [1,2]. Their use for promoting and maintaining a good state of health and the treatment of diseases has been around since ancient times in Asian regions, while in the West, this approach is considerably more recent. Medicinal mushrooms (MMs) are reported to have numerous pharmacological actions such as antimicrobial, anti-inflammatory, immunomodulatory, antidiabetic, cytotoxic, antioxidant, hepatoprotective, anticancer, antioxidant, antiallergic, antihyperlipidemic, and prebiotic properties, among others [2,3,4,5]. These activities are attributable to many bioactive metabolites present in the mycelium but above all in the fruiting body, whose biological effect varies according to the chemical nature and whose distribution varies according to the fungal species. A great deal of research has been carried out and is increasingly being undertaken to identify and characterize mycochemicals and to define their actions and mechanisms, due to the growing interest in the use of natural products, including as adjuvants in traditional therapies. The bulk of research carried out has focused on a few genera or species, for example, those of the oldest and most traditional use among Asian populations, while for those remaining, current scientific support is still lacking. Several studies have so far investigated the various activities of MMs, highlighting their enormous potential for use in the medical sector, but a particular effort has been put into studying their antitumor and immunomodulatory properties, as cancer remains one of the most difficult challenges to date.

The pharmacological activities of a medicinal fungus are detected primarily by in vitro assays, generally accompanied or followed by in vivo studies in animal models, which together reveal the great potential of a mushroom, fungal extract, or chemical compound. On the other hand, a small number of clinical studies carried out on humans and published in the peer-reviewed literature are available. Clinical studies are necessary to assess the efficacy of medicinal mushrooms within the complex human body system but also to assess their safety [2,5,6].

This review aims to provide a discussion of some of the best-known and most studied bioactive metabolites of medicinal mushrooms. In particular, we highlight the mechanisms of action by in vitro and preclinical scientific studies.

## 2. Bioactive Compounds in Medicinal Mushrooms: Effects and Mechanisms of In Vitro and In Vivo Preclinical Studies

As mentioned above, fungal compounds with bioactivity, and which are potentially useful for the prevention and treatment of various diseases, are very diverse.

The most important are polysaccharides, structural components of the fungal cell wall. The polysaccharides have a strong ability to carry biological information. More specifically, they have antitumor, immunomodulatory, antioxidant, anti-inflammatory, antimicrobial, and antidiabetic activity. In reality, the type and modulation of these biological activities are influenced by the specific structural features of the molecule, such as the weighted degree of branching, backbone linkage, side-chain units, and the type of constituent monosaccharides. The best known and most abundant are α- and β-glucans. Heteroglycans, peptidoglycans, and polysaccharide–protein complexes also contribute to biological activity [7,8,9]. They are primarily responsible for immunomodulatory effects due to their ability to bind to specific cell wall receptors and stimulate specific immune responses. Medicinal mushrooms are usually used in cancer treatments as biological response modifiers (BRMs), useful for treating cancer, reducing the side effects of therapies, and improving the quality of the patient’s life [1,3].

Another class of compounds that are very important for their bioactivity are the terpenes, characterized by units of five-carbon isoprene atoms and whose addition of functional groups produces the terpenoids. They modulate the immune system by stimulating the expression of genes coding for proteins involved in the immune response, but also have anti-inflammatory, antioxidant, and antitumor properties. High terpenoid contents are found in mushrooms belonging to the genus *Ganoderma* P. Karst. [2,7,9].

Mushrooms are rich in proteins, which have cytotoxic and anticancer properties. Some of them are known for their characteristic and marked immunomodulatory effect. These proteins are indicated as fungal immunomodulatory proteins (FIPs) whose mechanisms of action can be diverse [2,9]. Proteins also include lectins, which bind reversibly to mono- and oligosaccharides with high specificity, recognizing and interacting with various carbohydrates and proteoglycans on the cell surface. They are involved in many biological activities, such as innate immunity and cell-to-cell interaction, and their immunomodulatory mechanism varies depending on the origin of the compound. They also have immunomodulatory, antitumor, and antiproliferative properties [7,9].

Other fungal metabolites with bioactivity are phenolic compounds, antioxidants with different mechanisms of action (oxygen scavenging, metal inactivation, free radical inhibition, peroxidase decomposition), laccases (copper-containing oxidases), and fatty acids [2].

### 2.1. Coriolus versicolor (L.) Quél.

*Coriolus versicolor*, commonly known as tunzhi in China or turkey tail, has always been used as a “magic herb” in Asian regions and particularly in China, where ancient formulations based on this mushroom have been and are still widely used to promote good health, strength, and longevity [10,11]. Thousands of years ago in China, its medicinal properties were reported in the “Compendium of Materia Medica” and “Shen Non-Compendium Medica”, and currently in China, since 1987, and Japan, since 1977, *C*. *versicolor* extracts have been approved in routine clinical practice, especially in integrated cancer therapy in conjunction with chemotherapy or radiotherapy [10,11,12]. In China, there are currently at least 12 *C*. *versicolor*-based drugs approved by the State Administration of Food and Drugs (SAFD) for clinical use. The immunomodulatory properties of this mushroom are due to two protein-bound polysaccharides present in the fungal extract: the polysaccharide peptide (PSP), extracted from the deep layer cultivated mycelia of the COV-1 fungal strain and used most extensively in China, and the glycoprotein PSK (krestin), derived from the strain CM101 and used most widely in Japan. They are mainly composed of β-glucans and are among the most studied mushroom bio-compounds.

PSP extraction is done by boiling mycelia or even basidioma in water and further precipitation in ethanol. This protein-bound polysaccharide has a molecular weight of approximately 100 kDa, has a polysaccharide-to-peptide balance of 90–10%, is highly water soluble, and its carbohydrates are mannose, xylose, galactose, and fructose. [11]. PSP possesses immunomodulating, antitumor, anti-inflammatory, and antiviral effects, as reported by several in vitro and in vivo studies and some clinical trials; it has also shown other physiological effects, such as liver-protecting, system-balancing, antiulcer, antiaging and learning, and memory-enhancing properties, as well as reducing adverse events related to chemotherapy and radiotherapy treatments [10,12]. The immunomodulatory activity is due to the ability to a) act on cytokine release, b) increase the expression of cytokines and chemokines such as tumor necrosis factor-α (TNF-α), interleukins (IL-1β and IL-6), histamine, and prostaglandin E, c) activate natural killer (NK) cells and enhance dendritic and T cell infiltration into tumors. These actions are attributable to the β-glucan component of the polysaccharide, as these compounds are known to activate various immune cells expressing the corresponding receptors (such as dectin-1, toll-like receptors TLR-2, TLR-4, and TLR-6, and CR3 complement receptors) [11,12]. PSP induces apoptosis in human promyelocytic leukemia HL-60 cells by lowering the Bcl-2/Bax ratio and the mitochondrial transmembrane potential, releasing cytochrome c and activating caspase-3, -8, and -9 [13].

Studies in vitro or in mice models have shown an increase in lymphocyte proliferation and immunoglobulin IgG levels, suggesting effects on humoral immunity by PSP. Other results suggest a role for PSP in the activation of various pattern recognition receptors (PRRs), fundamental, therefore, in the innate immune response upon an encounter with a pathogen-associated molecular pattern (PAMP) [11]. One type of PRR whose specific level of action is a function of the infecting pathogen is toll-like receptors, which represent the body’s first line of defense and on which the PSP can act positively. Antiviral action of this kind was demonstrated in vitro by Rodríguez-Valentín et al. [14], showing that PSP not only downregulated viral replication and promoted the upregulation of specific antiviral chemokines, such as RANTES, MIP-1α/β, and SDF-1α, with blocking action on HIV-1 coreceptors in THP1 cells and human peripheral blood mononuclear cells (PBMCs), but upregulated TLR4 expression. A study lead by Wang et al. [10] in mice carrying a defective or normal TLR4 gene recorded how PSP stimulates the expression of both cytokines and TLR4 and its downstream signaling molecule TRAF6 and increases the phosphorylation of the transcription factor NF-κB p65 and the activator protein AP-1 transcription factor component c-Jun in peritoneal macrophages from TLR4^+/+^ mice, but not from TLR4^-/-^ mice. This shows that PSP-mediated immunomodulatory action occurs via the TLR4 signalling pathway. Besides, a reduction in tumors was also found compared to normal saline treatment.

Many in vitro studies in different models (human PBMCs from healthy or cancer subjects, murine splenic lymphocytes, primary mouse peritoneal macrophages, etc.) have shown that PSP treatment induces higher levels of TNF-α and the cytokines associated with it and IL-1β (the proinflammatory signal that enhances lymphocyte proliferation), but also IL-12 (enhancer of NK and CD8+ T cell activity and inducer of interferon IFN-γ), IL-6, and Il-1α, affecting the expression of many other pleiotropic cytokines, e.g., transforming growth factor (TGF)-β (proinflammatory effects on monocytes and Th17 cells; anti-inflammatory effects on B cells and regulatory T cells (T(regs)) and the activation of macrophages, and it also induces superoxide dismutase (SOD) and increases the sensitivity of immune cells to other stimuli [11,12].

PSK is a proteoglycan of about 100 kDa, with a polysaccharide-to-peptide balance of 40–60%. The carbohydrates are mannose, xylose, galactose, arabinose, and rhamnose [11]. Since it is also composed mainly of β-glucans, its activities and mechanisms are similar to PSP, again demonstrated in vitro or in mice models. Krestin showed both direct and indirect cytotoxic effects on cancer cells in vitro. As shown by Lu et al. [15] in their in vitro study on splenocytes from *neu* transgenic mice and in mice carrying a defective TLR2 gene, the antitumor action of this compound is mediated by TLR2 in mice. It increased dendritic cells (DC)s and CD4+ and CD8+ T cells, decreased B cells, induced the secretion of Th1 cytokines and IL-2, increased IFN-γ levels, and promoted DC maturation (CD86+ MHCII; higher levels of IL-12p40 and IL-12p70 were recorded, as was the inhibition of tumor growth. Other research has reported similar results, such as the activation of cytotoxic T cells (TC cells), improved DC maturation, the increased production of IL-8 and other cytokines (TNF-α, IL-1, IL-4, IL-6, IFN-γ) through the activation of T cell receptors (TCRs), enhanced major histocompatibility complex (MHC) class I expression by tumor cells, the inhibition of tumor growth, and the in vitro reduction of tumor growth factor-β (TGF-β) [16,17,18]. Thus, PSK can strengthen the body’s natural immune response. In a study on mice [19], this molecule was also shown to improve insulin resistance and hyperlipidemia by regulating the expression of inflammatory cytokines; a reduction in plasma triglycerides (TGs) and free fatty acids, the downregulation of the proinflammatory factors IFN-γ, IL-6, and IL-1β, and the upregulation of the expression of the anti-inflammatory factor IL-10 were observed. Therefore, PSK could be an important adjuvant in the management of cardiovascular risk related to hyperlipidemia.

Despite the many results obtained to date, the mechanisms of action of these two protein-bound polysaccharides are still not fully understood. The peptide component may also play a role in the bioactivity of PSK and PSP.

### 2.2. Ganoderma lucidum (Curtis) P. Karst.

Long known as the “mushroom of immortality”, *Ganoderma lucidum*, also known as ling zhi or reishi, is one of the most widely used medicinal mushrooms in the world today. It has been used to promote well-being and longevity since ancient times in traditional Chinese medicine, as it was included in Shen Nong’s Materia Medica (206 BC-8 AD), and it is now listed in the American Herbal Pharmacopoeia, Chinese Pharmacopoeia, and Therapeutic Compendium, and is also widely used as an adjuvant in the treatment of various types of cancer [20]. More than 100 reishi-based products are currently marketed, such as the nutraceuticals Ganopoly and Immunlink MBG, containing aqueous polysaccharide fractions, as well as a wide range of supplements often also containing other mushroom species extracts, functional foods, mycopharmaceuticals, and cosmeceuticals, prepared from carpophores, mycelia, or spore powder. *G*. *lucidum* is recognized for its numerous pharmacological properties, such as anticancer, hypoglycemic, immunomodulatory, antihypertensive, cytotoxic, anti-diabetic, antioxidant, antihyperlipidaemic, antimutagenic, antiaging, antimicrobial, and hepatoprotective properties, and many others. These properties are mainly due to two major groups of metabolites present in *G*. *lucidum*: triterpenes/triterpenoids and polysaccharides. Triterpene compounds are derivatives from lanosterol, including ganoderic acids, ganodermic acid, ganodermic alcohols, lucidones, and lucinedic acids, and they possess marked antitumor, antimetastatic, cytotoxic, and enzyme inhibitory properties. The main polysaccharides are α-1,3, β-1,3 and β-1,6-D-glucans and ganoderan with glucose as a major sugar component, characterized by a strong antiangiogenic and immune system-strengthening properties [20,21]. These two categories of molecules are primarily responsible for the anticancer properties of reishi, both by suppressing cell proliferation, metastasis, and invasion and by promoting apoptosis, combined with its immunomodulating, immunostimulating, antioxidant, and anti-inflammatory activities.

In particular, concerning immunomodulatory action, it has been observed that this occurs through multiple mechanisms, such as the activation of cytotoxic T cells, B lymphocytes, dendritic cells, macrophages, NK cells, the TLR-4 pathway, and other immune cells, as well as their by-products TNF-α, interleukins IL-1, IL-2, IL-3, and IL-6, and active nitrogen and oxygen intermediates [20]. Lee et al. [22] found that the *G*. *lucidum* triterpenes butyl ganoderate A and B and butyl lucidenate A and N exert an inhibitory effect on adipogenesis in 3T3-L1 cells. The first and last of these compounds exerted this action by suppressing the mRNA expression levels of fatty acid synthase (FAS) and acetyl-CoA carboxylase (ACC) genes. The researchers concluded that the inhibitory action of these triterpenes on 3T3-L1 cells is at least partly due to the downregulation of the adipogenic transcription factor sterol regulatory element-binding protein 1 (SREBP-1c) and its target Fanconi anemia (FA) group C gene (FAC) and ACC. Lucialdheydes A and C, ganodermanonol, and ganodermanondiol showed cytotoxic activity in vitro in Lewis lung carcinoma, sarcoma 180, T-47D, and Meth-A tumor cell lines [21]. An in vivo study on mice injected with inflammatory breast cancer (IBC) and treated with the commercial extract ReishiMax GLpTM (carpophore and cracked spores) highlighted a selective action on gene and protein expression, with smaller tumor size and weight and reduced expression of E-cadherin, mammalian target of rapamycin (mTOR), human eukaryotic translation initiation factor 4G (eIF4G), and p70 ribosomal protein S6 kinase (p70S6K) and the activity of extracellular regulated kinase (ERK 1/2) [8]. It has been observed in vitro that the antiproliferative and proapoptotic action of reishi terpenes, polysaccharides, and proteins occurs by stimulating the proliferation of undifferentiated spleen cells and the production of cytokines and antibodies. They also have an antimetastatic action, which they exert by activating the NF-κB and MAP kinase pathways, promoting cytokine release [20]. A great deal of research has been carried out on ganoderic acids; ganoderic acid T, for instance, has been shown to induce apoptosis in metastatic lung cancer cells by acting on the pathway linked to mitochondrial dysfunction and the expression of the tumor protein p53. Moreover, ganoderic acid D inhibited the proliferation of HeLa human carcinoma cells and induced G2/M cell cycle arrest and apoptosis [21]. Ganoderic acid DM has been shown in vitro to arrest osteoclastogenesis in bone marrow cells and RAW 264 cell D-clone (RAWD) by suppressing the expression of c-Fos and nuclear factor of activated T-cells c1 (NFATc1), with the consequent inhibition of dendritic cell-specific transmembrane protein (DC-STAMP) expression and reduced osteoclast fusion [23].

In addition to anticancer activity, cardioprotective activity was also reported by [24] in their preliminary study using transverse aortic constriction (TAC) in mice to model pressure overload-induced cardiomyopathy and treatment with spore oil; the treatment resulted in a normalized ejection fraction, corrected the fractional shortening generated by TAC and reduced left ventricular hypertrophy; analysis of total RNA expression revealed the reduced expression of genes associated with cardiac failure, as well as reduced levels of RNA circ-Foxo3.

Ganodermanondiol has also been reported by Kim et al. [25] for its inhibitory effect on melanogenesis by assaying its inhibitor effects on tyrosinase activity and melanin biosynthesis in B16F10 melanoma cells. The researchers pointed out the inhibition of activity and the expression of cellular tyrosinase, as well as of the expression of tyrosinase-related protein-1 (TRP-1), TRP-2, and microphthalmia-associated transcription factor (MITF), leading to reduced melanin production. The mitogen-activated protein kinase (MAPK) cascade and the cyclic adenosine monophosphate (cAMP)-dependent signaling pathway were also affected. These results make reishi the perfect candidate for the preparation of skincare products. An antiaging action of *G*. *lucidum* was shown in a recent study [26] by assaying the effects of ganoderic acid D on oxidative stress-induced stem cell senescence, using an H_2_O_2_-induced stem cell senescent model using human amniotic mesenchymal cells (hAMSCs) with a high expression of β-galactosidase, a senescence-associated marker. Ganoderic acid D inhibited the generation of reactive oxygen species (ROS) and senescence-associated markers, such as β-galactosidase, p21, and p16INK4a, and enhanced telomerase activity through the activation of the PERK/NRF2 signaling pathway.

*G*. *lucidum* possesses a plethora of other bioactive metabolites with numerous effects, more than 400 of which can be found in the literature (Bulam et al. 2019). In addition to those already seen, there are also peptides (GLP from the water-soluble extract, perhaps the main agent responsible for the fungus’s alternative oxidase (AOX) activity), peptidoglycans (GLPP, capable of neutralizing ROS damage in rat macrophages; ganoderan C with hypoglycemic activity), polyphenols, ergostan sterols and ergosterol, alkaloids, fatty acids with tumor proliferation-inhibiting action, nucleotides and nucleosides with platelet aggregation effects, the α-glucosidase inhibitor SKG-3, and laccase isoenzymes with antiviral properties [20,21].

### 2.3. Lentinula edodes (Berk.) Pegler

Lentinan, a β-1,3-D-glucan extracted from *Lentinula edodes* or shiitake, is another compound perdmitted in and widely used in Japan for the treatment of cancers, especially gastric cancer, due to its immunomodulatory action. Lentinan is a BRM capable of promoting Th1 response and improving Th1/Th2 balance. In several in vitro studies and in in vivo mice models, the polysaccharide activates dendritic cell function by increasing levels of tumor-infiltrating CD86+ cells, stimulates T and NK cell production, restores the killer/survival cell ratio, increases FcR receptor expression and thus promotes NK cell-mediated tumor cell killing, and increases IL-2 levels. The metabolite also appears to enhance complement-dependent cytotoxicity (CDC) and complement-dependent cell-mediated cytotoxicity via the CR3 receptor, known to be triggered by iC3b in fungal cell walls [27]. It can activate downstream signaling pathways, such as MAPK-NFκB and Syk-PKC, through binding to pattern recognition receptors such as TLR2/4/6/9 and Dectin-1, the complement receptor CD11b, and other membrane receptors, with the consequent activation of T cells, NK cells, and macrophages [28]. In both in vitro and in vivo studies, it has also been shown to increase the cytotoxic activity of primary macrophages and RAW264.7 cell lines, as well as cytotoxic activity and TNF secretion in macrophages, and cytotoxicity against sarcoma S180 cells by upregulating the proapoptotic protein Bax and downregulating the antiapoptotic protein Bc1–2, thus inducing apoptosis [28,29]. The action of lentinan is also expressed by interfering with cell cycles, as demonstrated in an in vitro study on rat C6 glioma cells [30]. The C6 cells’ activity was strongly inhibited in a dose- and time-dependent manner and the induction of apoptosis, cell cycle blockage, an increase in the proportion of cells in G0/G1 phase, and a decrease in those in S-phase were observed.

Lentinan is also able to act on the activation of inflammasomes, components of the innate immune system responsible for triggering inflammatory responses. This action is inferred from the results of a study conducted by Ahn et al. [29] on myeloid cells, involving mouse bone marrow-derived macrophages treated with lentinan with/without inflammasome triggers. Lentinan was found to selectively inhibit absent in melanoma 2 (AIM2) inflammasome activation, upregulate proinflammatory cytokines, and induce the expression of inflammasome-related genes through TLR4 signaling. Moreover, assessing the effect of lentinan on mice treated with *Listeria monocytogenes* or lipopolysaccharide as an AIM2 or non-canonical inflammasome-mediated model, the researchers found that the polysaccharide reduced IL-1β secretion due to activation of the *Listeria*-mediated AIM2 inflammasome, and also reduced endotoxin lethality by inhibiting the activation of the non-canonical inflammasome. An inhibitory effect of lentinan on tumor angiogenesis mediated by increased IFN-γ production was demonstrated in a study on the lung carcinoma cell line LAP0927 and colorectal carcinoma cell line CT26 [31]. The polysaccharide resulted in the upregulated expression of angiostatic factors and especially IFN-γ, as well as increased tumor infiltration of IFN-γ-expressing T cells and myeloid cells.

Underpinning the current use of lentinan as an adjuvant in oncological therapies are also several preclinical studies in which the polysaccharide was tested alongside substances used in chemotherapy treatment. For example, lentinan has been shown to alleviate the ROS-mediated nephrotoxicity of cisplatin (a key drug in lung cancer treatment) by activating the Nrf2-ARE signaling pathway. Similarly, a synergistic action with the tumor drug paclitaxel on A549 cells was exerted by activation of the ASK1/p38 MAPK signaling pathway and, consequently, of the thioredoxin-interacting protein (TXNIP)-associated NLRP3 inflammasome (TXNIP-NLRP3) [28].

An aqueous extract, particularly rich in polyphenols, of shiitake, was tested on human tumor cell lines of laryngeal carcinoma (Hep-2) and cervical adenocarcinoma (HeLa) for assessing its antiproliferative activity [32]. The extract displayed high free radical scavenging and catalase-like and cytotoxic activities, as well as the inhibition of cell proliferation and the induction of apoptosis as well as the *Pleurotus sajor-caju* (Fr.) Singer extract also tested in this study, albeit to a lesser extent.

### 2.4. Pleurotus spp.

Although not the best-known medicinal mushrooms, the *Pleurotus* species also have proven biological and effects. Several studies have been carried out to assess their antioxidative, antimicrobial, antidiabetic, anticancer, anti-inflammatory, immunomodulatory, antihypercholesterolemic, antihypertensive, antimicrobial, hepatoprotective, and antiaging properties, although the mechanisms underlying these effects have often not been elucidated, nor have the metabolites responsible been identified or characterized. The immunomodulatory and antitumor activities of *Pleurotus ostreatus* (Jacq.) P. Kumm. were reported by Sarangi et al. [33] by assaying, in vitro and in vivo, the effect of water-soluble proteoglycan fractions extracted from *P*. *ostreatus*, or oyster mushroom, on a sarcoma-180-bearing mouse model. Treatment with the mushroom resulted in a quantitative reduction of tumor cells and their arrest in the pre-G0/G1 phase of the cell cycle, increased cytotoxicity of NK cells, and the stimulation of macrophages to produce nitric oxide. In a study by Jedinak and Sliva [34] comparing the impact of different medicinal mushrooms on the growth of breast and colon cancer cells, *P*. *ostreatus* (PO) proved to be the most effective, suppressing cell proliferation via the p53-dependent and p53-independent pathways. More specifically, the methanolic extract of the mushroom induced the suppression of the proliferation of the human breast cancer cell lines MDA-MB-231 and MCF-7 and colon cancer cell lines HCT-116 and HT-29, and caused cell cycle arrest in the G0/G1 phase in MCF-7 and HT-29 cells. Furthermore, in MCF-7 cells, it induced the expression of the tumor suppressor p53 and the cyclin-dependent kinase inhibitor p21(Cip1/Waf1) and inhibited phosphorylation of the retinoblastoma protein Rb; the upregulation of p21 expression and inhibition of Rb phosphorylation was also observed in HT-29. A few years later, Jedinak et al. [35] demonstrated the anti-inflammatory properties of PO by testing a mushroom concentrate in vitro on the RAW264.7 murine macrophage cell line and murine splenocytes, in the absence or presence of lipopolysaccharide (LPS) or concanavalin A (ConA), and in vivo on Balb/c mice with LPS-induced inflammation. PO suppressed the LPS-induced secretion of TNF-α, IL-6, and IL-12 from macrophages and inhibited the LPS-induced production of prostaglandin E2 (PGE2) and nitric oxide (NO) through, respectively, the downregulation of the expression of COX-2 and iNOS, the suppression of the LPS-dependent activation of AP-1 and NF-κB, the suppression of the secretion of TNF-α and IL-6 in mice challenged with LPS in vivo, and the inhibition of ConA-induced splenocyte proliferation and the production of IFN-γ, IL-2, and IL-6.

A polypeptide (PEMP) extracted from *Pleurotus* *eryngii* (DC.) Quél. mycelium demonstrated significant free radical scavenging and antitumor activity in breast, cervical, and stomach cancer cells, whose growth it inhibited, and an activating effect on the macrophage-mediated immune response [36]. In a dose-dependent manner, it inhibited tumor cell proliferation, promoted macrophage proliferation and the expression of TNF- and IL-6 secretion, TLR2 and TLR4, and stimulated macrophage phagocytosis through the release of NO and H_2_O_2_. Cold-water extracts of *P*. *eryngii* var. *ferulae* (Lanzi) Sacc. and *P*. *nebrodensis* (Inzenga) Quél. showed marked in vitro anticancer activity on human HCT116 colon cancer cell lines [37]. Both treatments showed a marked inhibition of cancer cell viability and apoptosis induction, and a significant increase in the Bax/Bcl2 mRNA ratio; they also inhibited cell migration and affected homotypic and heterotypic cell–cell adhesion by inducing an increase in E-cadherin expression and negatively modulated the phosphorylation of both protein tyrosine and extracellular signal-regulated kinase ERK1/2.

A hot-water extract of *Pleurotus pulmonarius* (Fr.) Quél. has been shown to reduce in vitro and in vivo liver cancer cell proliferation and invasion by inhibiting the autocrine vascular endothelial growth factor (VEGF)-induced PI3K/AKT signaling pathway [8].

### 2.5. Grifola frondosa (Dicks.) Gray

*Grifola frondosa* or maitake is another major medical mushroom with numerous medicinal properties and whose main bioactive metabolite is the so-called D-fraction or GFP, a β-glucan proteoglycan compound. Several studies have demonstrated its antitumor effect, such as the one conducted by Alonso et al. [38] on MCF-7 human breast cancer cells. Not only did it activate macrophages, T cells, and NK cells, but it also triggered the expression of BCL2-antagonist/killer 1 (BAK-1) and several other genes (RASSF-2, FADD, IGFBP-7, ITGA2, ICAM3, SOD2, CAV-1, Cul-3, NRF2, Cycline E, ST7, and SPARC) involved in apoptotic stimulation, the inhibition of cell growth and proliferation and cell cycle arrest, the suppression of tumor cell migration and metastasis, and the downregulation of the PI3K-AKT signaling pathway. In a subsequent study in rodent LM3 mammary adenocarcinoma cells, it was observed that D-fraction, in addition to inducing apoptosis and reducing cell motility and invasiveness, increased cell adhesion by upregulating E-cadherin protein levels and inhibiting matrix metalloproteinase-2 (MMP-2) activity [39], while in triple-negative breast cancer (TNBC) cells, in addition to the effects just described, it also suppressed MMP-9 and reduced cell–cell adhesion by also increasing the membrane localization of the β-catenin protein [40].

Two polysaccharide fractions obtained from GFPs and named as F2 and F3 showed promising hypoglycemic effects in vitro, most likely due to enhanced insulin resistance stimulated by the reactivation of insulin receptor (IR) and insulin receptor substrate-1 (IRS-1); both fractions reduced levels of fasting serum glucose (FSG), fasting serum insulin (FSI), and the homeostasis model assessment of insulin resistance (HOMA-IR), and also enhanced IR and IRS-1 activities and the protein level of IR, reducing, on the contrary, that of IRS-1, and consequently acted on the PI3K/Akt pathways, as seen from the increased mRNA levels of PI3K and Akt [41].

Many other biologically active compounds have, however, been extracted from maitake and investigated for their effects. This is the case, for example, with a new glycoprotein extracted from the fermented mycelium of *G.* *frondosa*, GFG-3a. It can induce apoptosis in human SGC-7901 gastric cancer cells by acting on the stress response, p53-dependent mitochondrial-mediated, caspase-8/-3-dependent, and PI3k/Akt pathways [42]; apoptosis, cell cycle arrest at S phase, and the modulation of the expression of 21 proteins were observed and, in particular, the upregulation of 10 proteins, including RBBP4, associated with cell cycle arrest and the downregulation of 11 proteins, including RUVBL1, NPM, HSP90AB1, and GRP78, involved in apoptosis and stress response.

### 2.6. Hericium erinaceus (Bull.) Pers.

Very important and well-studied bioactive metabolites also include the erinacines (A-I), a group of cyathin diterpenoids extracted from the mycelium of *Hericium* *erinaceus* or lion’s mane or yamabushitake, and hericenones (C-H), benzyl alcohol derivatives extracted from the fruiting body. Both groups of compounds can easily pass through the blood–brain barrier and have demonstrated neurotropic and neuroprotective effects. They are reported to induce nerve growth factor (NGF) synthesis, both in vitro and in vivo. However, this medicinal mushroom also has antioxidative, anti-inflammatory, anticancer, immunostimulant, antidiabetic, antimicrobial, hypolipidemic, and antihyperglycemic properties, although its most frequent use is for the treatment of neurodegenerative diseases and cognitive impairment [4,43,44].

Erinacin A, the main representative of the erinacine group, has been proven to have an effective protective effect against Parkinson’s disease. In a 1-methyl-4-phenyl-1,2,3,6-tetrahydropyridine (MPTP) mouse model of Parkinson’s disease, erinacin A produced a reduction of MPTP-induced dopaminergic cell loss, apoptotic cell death induced by oxidative stress, and the levels of glutathione, nitrotyrosine, and 4-hydroxy-2-nonenal (4-HNE); it also reversed MPTP-associated motor deficits, and reduced the impairment of 1-methyl-4-phenylpyridinium (MPP)-induced neuronal cell cytotoxicity and apoptosis, through an endoplasmic reticulum (ER) stress-sustained activation of the IRE1α/TRAF2, JNK1/2, and p38 MAPK pathways, the expression of C/EBP homologous protein (CHOP), IKB-β, and NF-κB, as well as Fas and Bax [45]. This metabolite was also found to be effective against ischemic stroke, as reported in a study on rats in which the reduction of neuronal apoptosis, as well as the size of the stroke cavity in the brain by targeting iNOS/reactive nitrogen species (RNS) and p38 mitogen-activated protein kinase (MAPK)/CCAAT enhancer-binding protein homologous protein (CHOP) pathways, was observed [46].

Erinacin A was also reported to have significant antitumor activity in human gastric cancer TSGH 9201 cells, in which it induced significant apoptosis associated with increased phosphorylation of focal adhesion kinase/protein kinase FAK/Akt/p70S6K and serine/threonine kinase PAK-1 pathways. It also resulted in increased cytotoxicity and ROS generation, the reduced invasiveness and activation of caspases, and the expression of tumor necrosis receptor TRAIL [47]. The strong antitumor action of this metabolite was subsequently confirmed by a recent study both in vitro in two human colon cancer cell lines (DLD-1 and HCT-116) and in vivo in a mouse model [48] that further clarified its mechanisms. Treatment effects included stimulation of the extrinsic apoptosis activation pathways (TNFR, Fas, FasL, caspases), suppression of the expression of the antiapoptotic molecules Bcl-2 and Bcl-XL, and phosphorylation of Jun N-terminal kinase JNK1/2, responsive to stress stimuli, NF-κB p50 and p330. It was also demonstrated that the upregulation of death receptor molecules through the JNK MAPK/p300/NF-κB pathway is mediated by the modification of histone H3K9K14ac; the results of the in vivo assay revealed, in fact, increased levels of histone H3K9K14ac, as well as histone acetylation on Fas, FasL, and TNFR promoters.

Another erinacin, erinacin C, is known for its antineuroinflammatory and neuroprotective actions, which could be achieved through a mechanism of inhibition of IκB, p-IκBα (involved in the upstream NF-κB signal transduction cascade), and inducible nitric oxide synthase (iNOS) protein expression, and the activation of the Nrf2/HO-1 stress-protective pathway [49]. The treatment of human BV2 microglial cells with LPS-induced inflammation resulted in reduced levels of nitric oxide (NO), IL-6, TNF-α, and iNOS, the inhibition of NF-κB expression, and the phosphorylation of IκBα (p-IκBα) proteins, as well as the inhibition of Kelch-like ECH-associated protein 1 (Keap1), and increased nuclear transcription factor erythroid 2-related factor (Nrf2) and the expression of the heme oxygenase-1 (HO-1) protein.

### 2.7. Antrodia cinnamomea T.T. Chang and W.N. Chou

Anctin-A (ATA) is a bioactive steroid-like compound isolated from Antrodia *cinnamomea* or the AC mushroom, a little-known medicinal mushroom in the West, but very popular in Taiwan, where this endemic species is traditionally used to treat liver disorders resulting from alcohol intake and has many recognized therapeutic properties in addition to its hepatoprotective one. This mycochemical was reported to have antitumor effects on human MCF-7 and MDA-MB-231 breast cancer cells, in which it arrested epithelial-to-mesenchymal transition (EMT) processes by upregulating E-cadherin and occludin proteins and downregulating N-cadherin and vimentin proteins through suppressing their transcriptional repressor ZEB-1; it also caused induction of the ZEB-1 repressor miR-200c, associated with the transcriptional activation of p53, and the inhibition of cell motility and invasiveness [50].

The AC ethanolic extract also showed marked antiproliferative activity in human T47D breast cancer cells in vitro and in vivo. AC caused cell cycle arrest at the G1 phase, thus inhibiting proliferation, and induced autophagy. The reduced expression of cell cycle-related proteins and the increased expression of the transcription factor FOXO1, the autophagy marker LC3 II, and the protein p62 were recorded. The extract also mediated endoplasmic reticulum stress by upregulating the expression of inositol-requiring enzyme 1-α (IRE1), glucose-regulating protein 78 GRP78/Bip, and C/EBP homologous protein CHOP. Tumor growth was inhibited in vivo [51].

### 2.8. Agaricus bisporus (J.E. Lange) Imbach

*Agaricus bisporus* contain beta-glucans, ergosterol, ergothioneine, vitamin D, and flavonoids, with varying concentrations depending on the cooking method and duration, and UVB exposure [52]. Besides, essential amino acids, peptides, glycoproteins, nucleosides, triterpenoids, lectins, fatty acids, and their derivatives make this mushroom of considerable importance for its potential application as an antimicrobial, anticancer, antidiabetic, antihypercholesterolemic, antihypertensive, hepatoprotective, and antioxidant agent [53]. The consumption of *A*. *bisporus* in the diet is recommended to prevent prostate cancer due to the action of conjugated linoleic acid (CLA). Specifically, CLA inhibits proliferation in prostate cancer cell lines in vivo. The treatment showed an antiproliferative and proapoptotic action of mushroom extracts by inhibiting the growth of prostate cancer in athymic mice [54].

The nephroprotective effects of *P*. *ostreatus* and *A*. *bisporus* aqueous extracts on hyperoxaluria-induced urolithiasis induced in Wistar rats through the addition of 0.75% (v/v) ethylene glycol in drinking water for nine weeks have been investigated. The mushroom extracts inhibited the progression of nephrolithiasis and showed nephroprotective effects against ethylene glycol-induced kidney dysfunction [55].

The anti-inflammatory and antioxidant properties of *A*. *bisporus* biomass extracts from in vitro cultures were highlighted by Muszynska et al. [56]. The incubation of Caco-2 cells with *A*. *bisporus* extracts produced a decreased expression of cyclooxygenase-2 and prostaglandin F2α receptor compared with the LPS- and/or TNF-α-activated cells.

### 2.9. Agaricus blazei Murrill

*Agaricus blazei* contains several bioactive components which activate the immune system for a multitude of defensive functions [57].

Several animal studies and clinical experience have demonstrated that *A. blazei* possesses antitumor and immunological enhancement activity, and the fungus is also effective for the treatment of diabetes, HIV/AIDS, hypotension, and hepatitis [58].

β-glucans in *A*. *blazei* are the main constituents that stimulate the immune system and also act as antitumorals against myeloma and hepatic cancer in in vivo and in vitro studies [59,60].

Although further investigation of the efficacy of *A*. *blazei* extracts is required, some in vitro and in vivo preclinical studies have shown activity against Gram-positive and Gram-negative bacteria. In particular, the *A*. *blazei* mushroom extract promotes an antimicrobial effect against peritonitis, as well as deadly oral infections [61].

Another study highlights the clinical effect of the oral administration of *A*. *blazei* on the antibody response to β-glucan (anti-BG) titer [62]. The oral administration of *A*. *blazei* induced a β-glucan-specific response. The resulting anti-BG antibody production could be used as an index of the immune response to β-glucan in humans.

The influence of the β-glucans of *A*. *blazei* on the immune system can result in antiallergic effects. Such activities have been shown in vitro and in animal assays with an impact on the balance between Th1/Th2 cells in the immune system [63].

## 3. Mushroom Therapeutic Use: An Overview of Dietary Supplement Affairs

Mushrooms have been traditionally used for the maintenance of physical well-being and the treatment of numerous diseases since ancient times, especially in Asian regions. Since fairly recent times, they have become part of the sphere of dietary supplements widely employed for their health benefits, the use of which has largely entered into complementary alternative medicine (CAM) and complementary integrated medicine (CIM). Today, they are among the most commonly used of all integrative, complementary, and alternative therapies, especially in the field of oncology. This is especially the case in Asian countries, where mycotherapy has ancient and deep-rooted origins, while in Western areas, the application of mushrooms in medical therapies is still rather limited, especially in conventional medical institutions [5,20]. However, this is still a complex field in many respects. First of all, although a great deal of research has been carried out to highlight their various beneficial properties and thus their potential use in therapeutics, many mushrooms have only been tested in vitro or in vivo in animal models, mainly mice and rats, with little or no scientific support in vivo in humans [5,6]. Thus, although supplement companies often specify research to support their product claims, these are preclinical or even in vitro studies. Another aspect to take into account is undoubtedly the fact that there are innumerable mushroom supplements on the market today, but for the same species, doses, preparations, manufacturing practices, and claims vary considerably between manufacturers. In the absence of standardization, significant differences can be found even in different batches from the same manufacturer. This leads to considerable difficulties in the scientific practice of clinical trials, both because they are difficult to compare, but also and above all because there is no standardization of parameters such as dose, active ingredient/s, composition, adverse effects, and interactions, which ends up compromising the validity and repeatability of any results obtained and, consequently, their use in medical practice according to the criteria it requires.

It is precisely the heterogeneity, reduced quality, and lack of standardization of these mixtures that make it difficult for supplements to be considered by the Western medical community and integrated into conventional therapeutic practices [20]. However, there is a slow increase in the number of doctors using them in their daily practice. Another problem is the autonomous choices of people to take supplements, convinced of the efficacy of a “natural” substance.

Regarding claims about and labeling of dietary supplements, the American Food and Drug Administration (FDA) does not require manufacturers to prove safety and efficacy, although products must have a history of safety [64]. The European Food Safety Authority (EFSA) sets the rules for the use of nutritional health and disease risk reduction claims, requiring toxicological data; since 2011, a new “botanical” can only be registered as a food supplement and not as an actual drug, falling under EU Regulation No. 1924/2006 [65]. Therefore, for most mushroom-based supplements, safety and efficacy are generally supported by traditional use, in vitro studies, animal model studies, and some case reports.

It has to be said, however, that the increasing focus on these two attributes is resulting in more and more clinical investigative studies being carried out to prove them, albeit currently with the limitations mentioned above.

The lack of regulation and monitoring by many governments means that supplements are often not monitored to ensure that they contain the ingredients or the amount of active ingredient declared by the manufacturer. Indeed, unlisted components may be present, which may be either harmful or inert. Furthermore, the very fact that a fungal extract contains a multitude of demonstrably or potentially bioactive compounds often makes it difficult to link the effect to its true responsible agent, which also requires knowledge of the real concentration of the bioactive compounds contained in a supplement. Moreover, there is a risk that the consumer will not ingest the correct dose of an active substance, which may be higher, lower, or even non-existent [64]. In addition to all this, the presence of several compounds in the same supplement makes it difficult to carry out rigorous clinical studies. This is due to the complexity of identifying both the “optimal dose” of the preparation needed to guarantee the desired effect and the cause–effect relationship, since the different substances may act on several parameters at the same time and, moreover, in a synergistic or antagonistic manner.

In addition to all this, numerous other factors often limit the validity of the clinical studies conducted to date, even in the case of promising results. They often involve too small a sample (among other things, the enormous variability in sample size makes it difficult to compare the various studies), or lack a control or placebo group, or the two groups compared are numerically very different, there is a very frequent lack of replication, adverse events are poorly reported or investigated, the statistical methods are deficient, and the results are poorly described in various respects [5,6,9,66].

However, many clinical investigations have shown very encouraging or promising results, thus underlining the great potential of mushrooms in therapeutic applications.

## 4. Pharmacological Activities of Mushrooms: Medical Evidence through Clinical Trials

The effects of some fungal metabolites or extracts found to be effective in in vitro tests or preclinical research, and therefore of potential medical interest, have also been evaluated in vivo in human subjects in clinical trials.

### 4.1. Medicinal Mushrooms and Cancer Clinical Studies

An extract of *Agaricus blazei* Murrill Kyowa, known to have antimutagenic and antitumor properties, was used in a randomized clinical trial (RCT) of 100 patients suffering from various gynecological cancers (cervical, ovarian, and endometrial) and receiving chemotherapy [67]. The treated group showed increased NK cell activity, while no significant differences were found in lymphokine-activated killer and monocyte activities. Furthermore, administration of the fungal extract resulted in a clear reduction in chemotherapy-related side effects, such as loss of appetite, alopecia, emotional instability, and general weakness, as well as a marked improvement in mood-related parameters (anxiety, depression, mental stability).

Ineffectiveness, on the other hand, was the result of an open-label trial conducted in 2010 by Yoshimura et al. [68]. They compared two different supplements, Rokkaku Reishi containing *G*. *lucidum* and Senseiro containing *A*. *blazei* Murrill, and administered them orally for six months to a total of 51 prostate cancer patients following radical prostatectomy. The parameters assessed before and after treatment were serum prostate-specific antigen (PSA) level and PSA doubling time, with a partial response rate evaluated as the primary outcome; hormonal status (represented by serum testosterone levels) and toxicity were also evaluated. Both medicinal mushrooms had no significant cancer effect, showing no partial response in terms of PSA, and no correlation between PSA doubling time and serum testosterone levels.

Ascertaining the safety of an *A*. *blazei* Murrill treatment for cancer patients was the subject of a phase I clinical trial conducted by Ohno et al. [69]. Seventy-eight cancer survivors (30/24/24) were given one, two, or three packs of Senseiro (1800 mg/pack) daily for 6 months. Adverse events were defined by subjective/objective symptoms and laboratory data according to the National Cancer Institute Common Terminology Criteria for Adverse Events version 3.0 (NCI-CTCAE v3.0). Consumption of the mushroom proved safe in almost all patients. Only in nine cases were adverse events recorded, mainly digestive, such as nausea and diarrhea. Only one patient complained of a liver dysfunction-related food allergy, a drug-ymphocyte product. None of these adverse events occurred in a dose-dependent manner. No immune outcome was implemented.

An interesting case is Andosan^TM^ (ACE Co. Ltd. produced for Immunopharma, Gifu-ken, Japan), a product made from the mycelium of *Agaricus blazei*, as well as smaller amounts of *Grifola frondosa* (3%) and *Hericium erinaceus* (15%), three mushrooms traditionally used for therapeutic purposes in Asia. This product has been tested in various clinical trials, demonstrating antitumor, anti-inflammatory, and antiallergic action, presumably mainly due to β-glucans and isoflavonoids [70]. Although to date it remains unknown what the main constituent of this product is, as only small amounts of β-glucans have been detected in it, it seems that the results obtained in humans are due to the β-glucan stimulation of Peyer’s patches in the gut-associated lymphoid tissue (GALT), together with other less defined absorbable low-molecular-weight (LMW) substances, such as flavonoids [70]. In 2015, Tangen et al. [71] reported the results of a clinical trial in which Andosan^TM^ extract was orally given (60 mL/d) for seven weeks to patients with multiple myeloma undergoing high-dose chemotherapy with autologous stem cell transplantation (ASCT). In patients receiving Andosan^TM^, increased percentages of Treg cells (CD4+, CD127d+, and CD25+) and plasmacytoid dendritic cells (CD303+) were observed, as well as a significant increase in serum levels of interleukins IL-1ra (receptor antagonist), IL-5, and IL-7. Furthermore, gene expression studies were also carried out, showing increased expression of immunoglobulin genes, killer immunoglobulin receptor (KIR) genes, and Human Leukocyte Antigens (HLA) genes in the bone marrow in the *Agaricus* group; in more detail, an upregulation of endosomatic HLA genes and the plasma membrane CD86 gene was observed, and a downregulation of IL-7 and the proinflammatory chemokine CCL2 (MCP-1) genes, whereas the expression of the IL-5 gene was unaltered. In addition, the overall survival increased notably.

When the D-fraction (β-glucan) of *G*. *frondosa* was tested in patients with cancer (lung, lingual, breast, gastric, or liver cancer), inhibition of the progression of metastasis was observed and a reduced expression of tumor markers (carcinoembryonic antigen (CEA) and cancer antigen 15–3 (CA15–3) and CA19–9) [70]. The mechanisms underlying these effects are an increase in NK cell activity and Th1 response, besides a reduction in Th2 activity.

Andosan^TM^ has also shown anti-inflammatory effects in vivo in human subjects. In two different trials with healthy individuals, the ingestion of this drug provoked a reduction in proinflammatory cytokines, and an increased shedding expression of the adhesion molecule CD62L (L-selectin) and decreased intracellular reactive oxygen species (iROS), both in monocytes and granulocytes [70]. An improvement of quality of life was obtained by administering 60 mL of Andosan^TM^ for 21 days to patients with ulcerative colitis or Crohn’s disease, as well as a reduction in plasma levels of IL-5 and IL-2, respectively [58]. However, this study also found additional effects (improvement of total fatigue and/or lower cytokine levels) that have not yet been fully clarified.

The drug also appears to have antiallergic properties, as demonstrated in a study on patients suffering from birch allergy and asthma, where after administration of Andosan^TM^ before the pollen season, a clear improvement was observed, due to a reduction of specific IgE levels and mast cell sensitization, suggesting the potential use of mushroom polysaccharides as a substitute for corticosteroids [70].

Maitake’s effectiveness in fighting tumors was also highlighted in another study, in which patients with stage II-IV cancers were given mushroom powder or MD-fraction [72]. In high percentages of breast cancer patients (68.8%), lung cancer patients (62.5%), and liver cancer patients (58.3%), a cancer regression or a significant symptom improvement were observed, and the stimulation of immune-competent cell activities when the mushroom treatment was given alongside chemotherapy.

Interesting considerations were made by Deng et al. [73] following a phase I/II study with breast cancer patients who were given different doses of *G*. *frondosa* extract orally. They found that the resulting immunological effects were quite complex and varied according to the cell type and specific cytokine. Maitake intake seemed to have a stimulatory effect on some parameters but a suppressive one on others. In particular, the biggest functional changes were observed in granulocyte response to phorbol myristate acetate (PMA) stimulation, IL-10 production from CD14+ cells stimulated by PMA, IL-10 production from CD3+ cells stimulated by PMA, IL-2 production from unstimulated CD56+ CD3+ cell, and tumor necrosis factor- α (TNF-α) production from CD3+ cells stimulated by LPS, all parameters for which a medium dose (5–7 mg/kg) of the fungal extract was optimal, as well as for others such as IL-10 production from CD14+ cells stimulated by PMA, monocyte oxidative burst response stimulated by N-formyl methionyl-leucyl-phenylalanine (fMLP), and TNF-α production from unstimulated CD3+ cells. In contrast, for augmented granulocytes’ response to fMLP, augmented IFN-γ production from unstimulated CD45RO+ CD4+ memory T helper cells, and suppressed IFN-γ production from CD45RA+ CD4+ cells stimulated by PMA, the optimal dose was the highest one (10 mg/kg). This highlights that it is often difficult to find an “optimal dose” for botanical agents, probably because they generally contain several compounds that may act differently on a variety of target cells or with different strengths. Therefore, extreme caution should be exercised when treating individuals with major illnesses.

The dietary supplement based on maitake β-glucans, with recognized immunomodulatory properties, is also known to stimulate hematopoietic progenitor cell differentiation, granulocyte colony-stimulating factor production, and the recovery of peripheral blood leukocytes after bone marrow injury. Therefore, Wesa et al. [74] wanted to evaluate the effects of *G*. *frondosa* extract on 21 patients suffering from myelodysplastic syndrome (MDS) by giving them 3 mg/kg twice a day for 12 weeks and measuring the following parameters before and after treatment: neutrophil count and function, the latter tested as endogenous or stimulated neutrophil production of ROS by flow cytometry. *Escherichia* *coli*, the bacterial peptide fMLP, and phorbol ester were used as ROS activators. Maitake showed beneficial effects on MDS. It caused increased endogenous neutrophil and monocyte function and, compared to before treatment, an increased monocytic response to *E*. *coli* and fMLP-stimulated ROS production response were detected afterward.

Asian medical research has also focused on the two bioactive protein–polysaccharides extracted from *Coriolus* *versicolor* (L.) Quél., PSP and PSK or krestin, known for their immunomodulating and antitumor activities and which are widely employed. In a randomized, double-blind, placebo-controlled trial, subjects with advanced non-small cell lung cancer (NSCLC) who received PSP for 28 days following chemotherapy showed a significant improvement in blood leukocyte and neutrophil counts, serum IgG and IgM, and body fat percentage compared to the control group, resulting in a slower general deterioration [75].

Based on previous studies that suggested PSK could improve the survival of cancer patients when combined with chemotherapy, presumably through immunological mechanisms such as the induction of cytokines, regulation of Th1/Th2 balance, and inhibition of immunosuppressive molecules, Akagi and Baba [76] set out to test this product on advanced gastric cancer patients. Twenty-one subjects were randomly assigned to receive 300 mg tegafur/uracil (UFT) alone or 3g PSK together with 300 mg UFT daily for at least one year after surgery; immunological parameters were monitored and measured. Overall survival was markedly improved in the PSK group, with a 3-year overall survival of 62.2% compared with 12.5% in the untreated group. This result is possibly related to the observed reduction in CD57+ T-cells, known to indicate a poor prognosis in patients with advanced gastric cancer. Therefore, PSK is presumed to improve the overall survival of patients partly through the inhibition of CD57+ T cells.

Glycoprotein PSK was evaluated by Ito et al. [18] for its possible influence on the expression of major histocompatibility complex (MHC) class I expression in advanced gastric cancer patients after surgery. They compared two groups of subjects, one receiving only chemotherapy and the other immunochemotherapy with 3 g/day PSK for 19 months, in a total of 349 individuals. The two groups did not differ in their MHC class I expression. Nonetheless, within the PSK group, while MHC class I-positive patients had a relatively poorer prognosis than control patients, recurrence-free survival (RFS) was significantly higher in expression-negative subjects. Concerning the pN factor (number of lymph node metastases), expression-negative patients with pN2 or greater exhibited significantly higher RFS values. In contrast, for the factors pT (depth of tumor invasion) and venous invasion, no statistically significant differences were observed. The authors hypothesized the effectiveness of PSK adjuvant immunochemotherapy in MCH class I-negative patients and patients with advanced lymph node metastasis of pN2 or greater.

Despite the positive results of some studies and the common therapeutic use of PSK on tumors in Asian regions, several studies in this area have shown it to be ineffective. This is the case of the RCT carried out by Miyake et al. [77], in which they compared chemotherapy treatment based on UFT plus leucovorin (UFT/LV) with immunotherapy treatment with PSK implementation (UFT/PSK) in 357 stage IIIB or III colorectal cancer patients who had undergone Japanese D2/D3 lymph node dissection. Both the 3-year disease free-survival (DFS) and the 3-year overall survival (albeit with less marked differences) were lower in the UFT/PSK group, thus demonstrating the ineffectiveness, if not the disadvantage, of using this glycoprotein as an adjuvant to standard chemotherapy treatments in this type of patient.

UFT/PSK immunochemotherapy was also ineffective or even unfavorable in the 1-year randomized phase III trial performed by Okuno et al. [78] compared to surgery alone in 111 phase II colorectal cancer patients. Overall survival values were fairly similar in the two groups, while DFS was even worse in the treated group, although not statistically significant.

*C. versicolor* was tested by Chay et al. [79] in an RCT with 15 severe hepatocellular carcinoma (HCC) patients who had poor liver function or were unsuitable for standard therapy. A dose of 2.4 g/d of the extract was used. Compared to the placebo group, the mushroom-treated group had a higher median time to progression (TTP) (2.5 months vs. 4.2), median progression-free survival (PFS) (2.5 months vs. 1.1), and median overall survival (OS) (6.5 months vs. 2.2). A decrease in interleukin IL-17F and MCP-1, and an increase in prolactin and TNF-related apoptosis-inducing ligands were detected. Besides, there was an improvement, even if not statistically significant, in social, emotional, physical, and cognitive parameters, as well as a significant marked reduction in symptoms normally associated with disease progression, such as loss of appetite and pain. Therefore, this mushroom seems to be particularly promising for patients with palliation needs.

Powdered mycelium of *C*. *versicolor* was evaluated by Torkelson et al. [80] in a phase I two-center, dose-escalation clinical trial to work out the maximum tolerated dose when taken daily in divided doses for 6 weeks by women with breast cancer after standard chemotherapy and the recent completion of radiotherapy. Nine participants were assigned to three cohorts to take 3, 6, or 9 g of mushroom preparation (500 mg lyophilized mycelial powder/capsule). All three doses were well tolerated. Only nine adverse events were detected, seven mild, one moderate, and one severe (anxiety, probably unrelated to the treatment). The effects on the immunological system were also positive, with an increase in lymphocyte counts at 6 and 9 g/day, increased natural killer cell functional activity at 6 g/day, and a dose-related increase in CD8+ T cells and CD19+ B cells, unlike CD4+ T cells or CD16+56+ NK cells. Although this study showed the safety of *C*. *versicolor* at a 9 g daily dose, did not determine the safety and tolerability of higher doses, i.e., the maximum dose tolerated (MDT), and the sample size was very small.

*Ganoderma lucidum* or reishi is one of the oldest medicinal mushrooms used in Asian regions, and is consequently one of the most studied up to the present day. About 400 bioactive compounds have been isolated from the mycelium, fruiting bodies, and spores of this mushroom, including polysaccharides, triterpenes, ganoderic acids, phenols, amino acids, lignin, vitamins, nucleosides, nucleotides, sterols, steroids, proteins, unsaturated fatty acids, and inorganic ions [81]. However, the most important bioactive molecules turn out to be polysaccharides, the most important of which are β 1–3 and β 1–6 D glucans, and triterpenes, especially the lanostane type. They have shown multiple important therapeutic properties both in vitro in numerous tumor cell lines and in vivo in animal models, thus explaining why Chinese medicine traditionally uses extracts of this mushroom for cancer prevention and treatment. They are also used to alleviate the side effects of radio- and chemotherapy.

A randomized, double-blind, placebo-controlled, multicenter clinical trial was performed by Gao et al. [82] on 68 patients suffering from advanced lung cancer to assess whether *G*. *lucidum* had beneficial effects on the subjects’ quality of life. In individuals taking 600 mg/d of Ganopoly^®^ (Alpha s.r.l., Lodi, Italy) (a polysaccharide product from *G*. *lucidum*) three times daily for 12 weeks, a marked improvement in the quality of life was observed, as demonstrated by significantly higher Karnofsky scores than in the placebo group. Quality of life in general also improved significantly, regarding disease-related symptoms such as fever, weakness, insomnia, sweating, and coughing. Ganopoly^®^, administered at doses of 1800 mg/d three times daily for 12 weeks in 34 advanced lung cancer patients, also had positive effects on immunological functions, producing significant increases in plasma IL-2, IL-6, and IFN-γ concentrations, Cd56+, phytohemagglutinin (PHA) responses, and NK activity, as well as significant decreases in IL-1 and TNF-α [83].

Back in 2010, Oka et al. [84], wishing to confirm the cancer-preventive action of the water-soluble extract of *G*. *lucidum* mycelia (MAK) in vivo, conducted a no-treatment concurrent controlled study with 123 patients with colorectal adenomas who were disposed to undergo colonoscopy. They were given 1.5 g/d MAK for 12 months, after which they underwent a colonoscopy to detect the size, location, and macroscopic type of adenomas, then the data were compared with the untreated control group. In contrast to the untreated group, there was a significant decrease in both the number and size of adenomas in the individuals taking MAK, suggesting a suppressive effect of the extract on the development of colorectal adenomas and precancerous lesions of the large bowel.

Two years later, Zhao et al. [66] published the results of a pilot randomized clinical trial with 48 breast cancer patients with cancer-related fatigue undergoing endocrine therapy receiving 1000 mg of *G*. *lucidum* spore powder three times a day for 4 weeks. The survey parameters were obtained using the Functional Assessment of Cancer Therapy: Fatigue (FACT-F), Hospital Anxiety and Depression Scale (HADS), and European Organisation for Research and Treatment of Cancer Core Quality of Life Questionnaire C30 (EORTC-QLQ-C30) questionnaires. The concentrations of TNF-α and IL-6 and liver–kidney functions were assessed both before and after treatment.

Compared to the control group, significant increases in overall quality of life and physical function were observed in treated individuals, as well as an equally significant improvement in emotional and functional well-being, state of depression and anxiety, emotional functioning, cognitive function, and symptoms like fatigue, sleep disturbance, and appetite loss.

Besides, significantly lower levels of TNF-α and IL-6 were found in the serum. Thus, the spore powder exerted its anticancer and immunomodulatory effect without causing any serious adverse effects on individuals. Additionally, no alterations in kidney and liver function were found in the corresponding tests; only moderate discomfort, such as dizziness and dry mouth, occurred in a small percentage of the treated group.

The anticancer effect of *G*. *lucidum* glucans is attributable to immunological mechanisms, using the activation of T lymphocytes and macrophages, resulting in cytokine release [81] and the activation of B lymphocytes and NK, dendritic, and other immune cells, together with their secretory products like tumor necrosis factor-α (TNF-α), reactive nitrogen, oxygen intermediates, and interleukins (IL-1, IL-2, IL-3, IL-6) [20].

The inhibition of angiogenesis is also thought to be a mechanism involved in antitumor effects. A reduction in the mortality rate in cancer patients following the administration of reishi products (4–6 g/d for 2 months), or an immunological enhancement (capsules of 1800 mg/d for 12 weeks), and a general improvement in the quality of life have been observed in various clinical studies [69]. According to recent experimental evidence, *G*. *lucidum* inhibits the motility of tumor cells, interfering with the signaling pathway and thus having very important consequences on the spread of metastases.

In addition, *Pleurotus cornucopiae* (Paulet) Rolland or tamogitake was associated with the upregulation of the immune system in a double-blind placebo-controlled clinical trial by Tanaka et al. [85], suggesting its great potential for the prevention of various diseases, such as cancer and infectious diseases. This study showed that the administration of a fungal extract for eight weeks increased interferon IFN-γ and IL-12, and a smaller increase in NK cell activity, while serum levels of Th2-type cytokines IL-10 and IL-13 and other cytokines remained unchanged. The enhancement of the immune system by tamogitake appears to occur through Th1 phenotype potentiation and the macrophage–IL-12 and IFN-g pathway, thus resulting in activation of the cell-mediated immune system, but with almost no change in Th2-type cytokines (the causes of which have yet to be clarified).

*Agaricus bisporus* (J.E. Lange) Imbach showed promising results against prostate cancer by reducing immunosuppressive factors. In phase I, a single-arm, unblended, single-facility trial involving 36 patients, Twardoski et al. [86] observed that, following the administration of tablets of fungal extract (six mushroom dose cohorts: 4, 6, 8, 10, 12, and 14 g/day), there was a decline in PSA level from the first few months of intake until it became undetectable. At the same time, there was an increase in baseline IL-15 levels, and declines in myeloid-derived suppressor cells (MDSCs). The maximum dose level was capped at 14 gm daily, which was the highest dose that was thought to be practical to ingest over a long period of time, based on the number of tablets required.

Another species, *A*. *sylvaticus* Schaeff., was tested by Costa Fortes et al. in a randomized, double-blind placebo-controlled clinical trial with 56 patients with colorectal cancer in the post-surgery phase [87,88]. A mushroom extract (tablets) in doses of 30 mg/kg/d or placebo was administered to the two subgroups for six months, monitoring their metabolic, biochemical, and enzymatic profile, as well as parameters describing quality of life. In contrast to the placebo group, several significant positive effects were found in the treated group, such as the reduction of fasting plasma glucose level, reduction of total cholesterol, reduction of serum thyroglobulin and creatinine levels, decreased aspartate aminotransferase (AST) and alanine aminotransferase (ALT) and also of IgA and IgM, and the reduction of diastolic blood pressure (DBP), whereas total protein content and globulin increased. Regarding quality of life, the treated group showed improvements in various aspects (quality of sleep, appetite, pain, mood, and gastrointestinal symptoms).

The effects of *A*. *sylvaticus* on the side effects of chemotherapy in cancer patients were also investigated by Valadares et al. [89] in a randomized, double-blind, placebo-controlled trial of 46 women with breast cancer administered 2.1 g/d of the extract for 6 months. Again, there was an improvement in the patients’ condition in various respects, such as pain, appetite, and bowel function, with a very low rate of the occurrence of certain side effects compared to the placebo group.

In the RTC of Tsai et al. [90], *Antrodia* *cinnamomea* T.T. Chang and W.N. Chou, a popular medicinal mushroom in Taiwan, was tested in subjects suffering from different kinds of cancer (gastric, lung, breast, liver, or colorectal) alongside chemotherapy. A total of 37 patients were randomly assigned to receive either placebo or 20 mL of an *A*. *cinnamomea* (AC) oral formulation (mainly containing polysaccharides, triterpenoids, and γ-aminobutyric acid) twice a day for 30 days. Although lower mortality rates and longer overall survival were found in the mushroom-treated group than in the placebo group, these differences were not statistically significant.

The EORTC-QLQ-C30 also showed no substantial differences between the two groups, except for better sleep quality in the AC group. It is further important to remark that, even if most hematologic, liver, or kidney functions did not differ significantly between the two groups, a significant reduction in platelet count was observed in the AC group at the end of treatment. The study also reports a number of adverse effects in both samples, the most recurrent of which were gastrointestinal complaints, which were even more frequent in the AC group, albeit of a lesser severity, than in the control. Although this medicinal mushroom has traditionally been widely used in Taiwan for the treatment of various cancers, the results of this trial provide no evidence of its efficacy in improving the outcome of patients with advanced cancer. The authors speculate, however, that this may be due to the limited sample size, its short duration, and, not least, the inclusion of subjects with different cancer types.

In 2011, Ina et al. [91] wanted to test whether the biological response modifier (BMR) lentinan, used in combination with oral fluoropyrimidines (S-1 chemotherapy), could be effective in patients with gastric cancer. For this purpose, 78 subjects with metastatic or recurrent gastric cancer undergoing S-1 chemotherapy were selected, a proportion of whom were given 2 mg/body weight of lentinan intravenously for 30 min every 2 to 3 weeks. Immunochemotherapy with lentinan has been shown to significantly increase the overall survival of patients with advanced gastric cancer, although without consistent differences in the incidence and degree of adverse effects. Another randomized clinical trial was reported by Ma [92], comparing the effect of lentinan when combined with nanoparticle (NP) chemotherapy (cisplatin plus navelbine) on advanced non-small cell lung cancer patients. A significantly lower Parkinson disease (PD) rate and significantly higher efficiency rate and control rate were observed in treated subjects compared to the control group, as well as higher levels of CD3+, CD4+, CD8+, and CD4+/CD8+, showing that the immunochemotherapy with lentinan is able to improve the therapeutic efficacy, enhance immune functions, and reduce the adverse effects.

In addition, a more recent clinical study by Wang et al. [93] showed that lentinan-based chemoimmunotherapy seems to be a promising strategy for antitumor activity via enhancing the proliferation of cytotoxic T cells (CD3+ CD8+) and CD3+ CD56+ NKT cells, followed by the elevation of proinflammatory chemokines/cytokines (IFN-γ, TNF-α) and proinflammatory IL-12, which would then lead to a shift in the Th1/Th2 balance towards Th1. These were the results observed in subjects treated with 4 mg/d of lentinan for 12 weeks in relation to the total number of patients with NSCLC involved in the trial.

From the mycelium of the medicinal mushroom *Lentinula edodes* (Berk.) Pegler, or shiitake or ling zhi, active hexose correlated compound (AHCC), a nutritional supplement containing polysaccharides (including α-1,4-glucans), amino acids, and minerals, has been obtained [94].

Various studies report this product to have beneficial effects in the treatment of cancer, as it acts as an immunoenhancer able to alleviate the adverse effects of chemotherapy. In 2014, Ito et al. [95] performed a clinical trial with 24 cancer patients undergoing chemotherapy, and showed that the administration of AHCC significantly decreased the levels of herpes virus type HHV-6 in saliva (generally increased by chemotherapeutic treatments), improving quality of life and also chemotherapy-associated hepatotoxicity and hematotoxicity in the cancer patients.

Additionally, Del Buono et al. [96] performed a small clinical study to assess the effectiveness of AHCC for integrative cancer treatment, evaluating its immunomodulatory effects on the lymphocyte population.

A dose of 3 g/d of AHCC was given for 1 month to seven patients suffering pancreatic, lung, or colorectal adenocarcinoma. At the end of treatment, there were consistent increases in neutrophils and the population ratios of CD3/CD4, CD4/CD8, CD8/CD3 (suppressor/cytotoxic), and CD3+/CD16+/CD56 NK cells, while lymphocytes and monocytes decreased. Thus, α-glucans were more effective on innate immunity (CD8, CD56) than acquired immunity (CD4), and exerted a marked modulation of NK cells, which are crucial for the direct destruction of neoplastic cells. No toxicity was observed.

Positive results for AHCC were also obtained in a clinical study conducted with patients suffering from epithelial ovarian cancer or peritoneal cancer undergoing platinum-based chemotherapy [94]. An intake of 3 g/d (500mg/capsule) throughout six cycles of chemotherapeutic treatment resulted in increased levels of CD8+T cell lymphocytes, and in reduced side effects, such as nausea and vomiting. However, increased muscle pain was noted.

D’Orta et al. [97] also showed the beneficial effects of AHCC on sarcopenia in patients suffering adenocarcinoma and malnutrition undergoing radio-chemotherapy, by giving 50 of them a food therapy and 1.5 g/d of AHCC for 6 months. In 80% of subjects, no progression of cancer malnutrition or cachexia was observed, but rather an increase in body cell mass.

### 4.2. Medicinal Mushrooms and Diabetes, Hyperglycemia, Hyperlipidemia, and Cardiovascular Disorder Clinical Studies

The efficacy of *A*. *blazei* Murrill in diabetes control was also demonstrated in a randomized, double-blind, placebo-controlled trial involving 72 individuals with type 2 diabetes treated with gliclazide and metformin for more than 6 months [98]. The *Agaricus blazei* mushroom(ABM) extract was administered for 12 weeks at a dose of 1500 mg/d and then the homeostasis model assessment for insulin resistance (HOMA-IR) was evaluated. The administration of the ABM extract was associated with a significant improvement in insulin resistance, probably due to an increase in plasma adiponectin concentration, as this parameter increased reasonably in the ABM group and decreased in the placebo group.

The beneficial effects of a diet based on *A*. *bisporus* have been evaluated in pregnancy-related complications, like hypertension and macrosomia, in a recent randomized, placebo-controlled clinical trial performed by Sun and Niu [99]. A sample of 1244 women planning for their first pregnancy was recruited and randomly assigned to consume at least 100 g of white button mushroom (cooked according to personal taste) daily or follow a normal diet, from the pre-pregnancy stage to the 20th week of gestation. In the end, 582 women in the WBM group and 580 in the placebo group completed the program. The primary outcome was gestational hypertension, measured by diastolic blood pressure (DBP) and systolic blood pressure (SBP); the secondary outcome included preeclampsia, gestational body weight gain, other pregnancy complications such as gestational diabetes mellitus, and birth weight. The results showed a beneficial impact of the mushroom diet on all the parameters mentioned above, and a significantly lower risk of gestational hypertension, preeclampsia, gestational weight gain, gestational diabetes, and macrosomia was observed.

No significant positive results were obtained by Klupp et al. 8 [100] when they investigated the effectiveness of reishi in treating hyperglycemia and cardiovascular risk components of metabolic syndrome through a prospective, double-blind, randomized, placebo-controlled trial. For this purpose, they selected a total of 84 subjects with type 2 diabetes mellitus (DMT2) and metabolic syndrome and randomized them to receive 3 g/d of *G*. *lucidum* alone, *G*. *lucidum* combined with *Cordyceps* *sinensis* (Berk.) Sacc, or placebo for 16 weeks. Blood glucose was measured in terms of glycosylated hemoglobin (HbA1c) and fasting plasma glucose (FPG), but also other parameters like blood pressure, triglycerides, waist circumference, body mass index (BMI), health-related quality of life, C-reactive protein, total High-Density Lipoprotein (HDL) and Low Density Lipoproteins (LDL) cholesterol, and apolipoproteins A and B. Surprisingly, at the end of the treatment, reishi had not significantly influenced any of the aforementioned factors, thus not supporting the hypothesis of its possible use in the treatment of metabolic syndrome.

The capacity of *Pleurotus* *ostreatus* to reduce blood glucose, cholesterol, and triglycerides in diabetic patients was investigated by Kathun et al. [101], while also checking for possible hepatic and renal toxicity. A clinical trial was conducted with 89 subjects consuming 50g of cooked mushroom thrice daily for 24 days, by alternating 7 days of the mushroom diet and 7 days of no mushrooms and measuring different parameters at the start and after every 7 days. The significant effects found in patients were the reduction of systolic and diastolic blood pressure (SBP, DBP), plasma glucose, total cholesterol (TC), and triglycerides (TGs), while there was no substantial change in weight and HDL. When mushrooms were not consumed, there were significant increases in DBP, FPG and PG 2 h after breakfast (2 hPG), TC, and TGs, while SBP, HDL, and weight did not change. On resuming mushroom intake, the changes described above occurred again. Therefore, this fungal species can provide important beneficial effects in diabetic patients, without compromising liver and kidney function.

Other studies followed in subsequent years, aimed at assessing the effects of ingesting *P*. *ostreatus* in the form of fresh, cooked, or dried mushrooms on conditions such as DMT2, dyslipidemia, hypertension, overweight, or obesity. Kajaba et al. [102], for example, showed a significant hypolipidemic effect of *P*. *ostreatus* in their uncontrolled clinical trial of 57 individuals with, primarily, combined types of dyslipidemia, following a daily administration of 10 g of freeze-dried and pulverized mushroom for 6 weeks, and found a significant decrease in TGs, TC, and TC/HDL-C (High-Density Lipoprotein Cholesterol). There was also a decrease in the concentration of conjugated dienes (CDs) in plasma, while glutathione peroxidase (GPX) activity and the concentration of glutathione (GTH) in erythrocytes increased.

The cholesterol-lowering properties of an oyster mushroom diet were investigated for the first time in humans by Schneider et al. [103]. In their RTC, 20 individuals with moderate untreated hyperlipidemia were randomly assigned to take either a soup containing 30 g dried oyster mushrooms or a tomato soup as a placebo daily for 3 weeks. In the mushroom-fed group, significant reductions in TG concentrations and oxidized low-density lipoprotein (oxLDL) levels were detected, as well as a significant trend towards a lowering of TC values.

Choudhury et al. [104] performed an uncontrolled trial to assess the effects of *P*. *ostreatus* on the blood pressure and glycemic status of 27 hypertensive diabetic DMT2 drug-treated males. Capsules with sun-dried powdered mushroom (3 g/d; thrice daily amounts of 1 g) were administered for 3 months. A significant reduction of both SBP and DBP was detected, as well as of FGP and glycated hemoglobin (HbA1c), resulting in an improved glycemic status and blood pressure control. Moreover, no adverse effect on renal function was detected, as demonstrated by no significant change of plasma creatinine level.

A further uncontrolled study was carried out by Choudhury et al. [105] to investigate the effect of oyster mushrooms on the lipid profile of obese or overweight hypertensive non-diabetic subjects. After administrating pulverized mushroom capsules at a dose of 3 g/d for 3 months to 14 patients, significant decreases in plasma TC and low-density lipoprotein cholesterol (LDL-C) levels were observed. TGs and HDL-C also decreased, although not significantly.

These results suggest that *P*. *ostreatus* might be prescribed to improve the atherogenic lipid profile, primarily TC and LDC-L, of obese hypertensive individuals. Additionally, Sayeed et al. [106] performed a clinical trial to evaluate the metabolic effects of *P*. *ostreatus* in diabetic DMT2 drug-treated subjects, randomly assigning 73 women to consume 200 g/d mushrooms or placebo (equal calorie diet of vegetables) for 1 year. Significant decreases of FBG, 2-hPG, TC, TG, and LDL were observed in the treated group, whereas no differences were detected among groups for BMI, SBP, DBP, HDL, Hb, ALT, and creatinine. Finally, the hypolipidemic effects of the oyster mushroom and *Pleurotus* *cystidiosus* O.K. Mill. were tested by Jayasuriya et al. [107] on both healthy individuals and dietetically treated DMT2 diabetics, by oral daily administration for two weeks of a suspension of one of the two freeze-dried and pulverized mushrooms at a dose of 50 mg/kg BW, followed by a glucose load. In healthy subjects, both fungal species showed a significant decrease in FPG and postprandial plasma glucose (PPG). Equally significant was the lowering of PPG in diabetic patients for both mushrooms, for whom an increase in postprandial serum insulin levels was also observed, thus indicating these mushrooms as beneficial functional foods for diabetes mellitus.

### 4.3. Medicinal Mushrooms and Neuron Health Clinical Studies

*G*. *lucidum* has been widely used in Asian regions to treat not only cancer but diabetes and neurasthenia as well. For the latter, the mushroom seems to have particularly beneficial effects, as demonstrated in a randomized, double-blind, placebo-controlled clinical trial conducted by Tang et al. [108] with Ganopoly^®^. A total of 132 neurasthenic patients were randomized to receive placebo or 1800 mg three times daily for 8 weeks. At the end of treatment, patients taking *G*. *lucidum* were found to have an increased sense of well-being as measured by the Visual Analogue Scale (VAS), and consistent reductions in the Clinical Global Impression (CGI) severity scale score and fatigue.

The beneficial effects of *Hericium erinaceus* have been demonstrated in numerous clinical studies. Mori et al. [109], for example, conducted a double-blind, parallel-group, placebo-controlled clinical trial with 30 middle cognitive impairment patients, giving them four 250 mg tablets containing 96% mushroom powder or placebo three times a day for 16 weeks, continuing the follow-up for a further 4 weeks and performing assessments using a cognitive function scale based on the Revised Hasegawa Dementia Scale (HDS-R).

Compared to the placebo group, at weeks 8, 12, and 16 of treatment, and 4 weeks of follow-up, the yamabushitake group showed significantly increased scores, although at the fourth week, after the end of ingestion, the values decreased significantly. Regardless, this fungal species has proved to be very useful in improving average cognitive impairment. Additionally, on the side of these promising results, Li et al. [110] wished to test the efficacy of this mushroom in the treatment of early Alzheimer’s disease. In this randomized, double-blind, placebo-controlled pilot study, three capsules of *H*. *erinaceusHE* (HE) mycelium (350 mg/capsule; 5 mg/g erinacin A as active ingredient) were administered daily to patients with early-onset Alzheimer’s disease for 49 weeks. When comparing these subjects with the placebo group, there were significant differences in the Instrumental Activities of Daily Living score, a significant improvement in the Mini-Mental State Examination score, and contrast sensitivity. Furthermore, whereas in the placebo group the mean apparent diffusion coefficient (ADC) values from the arcuate fasciculus region in the dominant hemisphere increased significantly, in the mushroom-treated group, this was not the case, and a significant decrease in ADC values from the parahippocampal cingulum region in the dominant hemisphere was detected.

Because of the effect of *H*. *erinaceus* (HE) on the autonomic nervous system and brain function, the effects of this mushroom on menopause, depression, sleep quality, and undefined disorders were investigated by Nagano et al. [111] in a randomized, double-blind, placebo-controlled trial. Evaluations were carried out according to the Kupperman Menopausal Index (KMI), the Pittsburgh Sleep Quality Index (PSQI), the Center for Epidemiologic Studies Depression Scale (CES-D), and the Indefinite Complaints Index (ICI). Thirty females were randomly assigned to consume either four HE cookies (0.5 g powdered carpophore per cookie) or four placebo cookies daily for 1 month. In the treated group, the CES-D and ICI scores after the HE intake were significantly lower than before; compared to the placebo group, the “insensitive” and “palpitation” terms of the ICI were significantly lower, and the terms “concentration”, “irritating”, and “anxious” tended to be lower. Finding that *H*. *erinaceus* can alleviate anxiety and depression, the authors were led to suppose a different mechanism of action from the more familiar NGF-enhancing action of the mushroom.

A case study was reported by Inanaga [112] about the treatment with HE of an 86-year-old subject with recurrent depressive disorder suffering mild cognitive impairment during antidepressant therapy with mirtazapine. After 6 months of the intake of HE extracts in the form of Amyloban^®^3399 (US patent pending. Mushroom Wisdom, Inc., East Rutherford, NJ, USA), both cognitive function and body weight were successfully restored. The supplement Amyloban^®^3399 was also evaluated by Okamura et al. [101] for the treatment of sleep disorders in eight female undergraduate students through the daily intake of six tablets for 4 weeks, measuring sleep quality and general well-being status by the General Health Questionnaire (GHQ-28) and PSQI. Anxiety and insomnia showed a decreasing trend, as well as the PSQI scores. Furthermore, after 4 weeks of mushroom intake, increased levels of salivary free 3-methoxy-4-hydroxyphenylglycol (free MHPG) were detected, with this considered an accurate index of chronic stress and depressive symptoms, thus reflecting sympathetic nervous system activity. Although this pilot study showed the ability of *H*. *erinaceus* to improve well-being, sleep disturbance, and negative mood, as recognized by the authors themselves, caution should be exercised in making conclusions, as the sample size was too small. Besides, the selected subjects were recruited in the month before a major national examination, so all complaints probably lacked the severity and chronicity of clinically diagnosed anxiety and mood disorders. A more recent randomized clinical trial was performed to assess how *H*. *erinaceus* can act on depression, anxiety, sleep disorders, and binge eating [113].

Seventy-seven subjects affected by overweight or obesity and with one or more mood disorders were randomized to receive three capsules of an HE dietary supplement for two months daily and under a low-calorie diet regimen. The supplement administered (“Mycotherapy Hericium”) consisted of 80% bulk mycelium and 20% fruiting body extract. Before treatment, after the first month, and after the second month, the abovementioned complaints were assessed by using the Symptom Checklist-90, Zung’s Self-Rating Depression Scale, Zung’s Self-Assessment Anxiety Scale, and Binge Eating Scale (BES). All evaluations revealed significant improvements in depression, anxiety, and sleep quality in the HE-treated group. Moreover, with reference to the serum balance between brain-derived neurotrophic factor (BDNF) and its precursor pro-BDNF, an increase in circulating pro-BDNF levels was detected, but without fully clarifying whether these neurotrophins can actually be used as biomarkers of mood disorders.

### 4.4. Medicinal Mushrooms in Clinical Studies on Other Medical Topics

In order to ascertain the optimal dose of *A*. *bisporus*, or white button mushroom (WBM), to achieve in humans the effects of suppressing aromatase activity and inhibiting breast cancer cell proliferation already established in vitro and in preclinical tests, Palomares et al. [114] carried out the following dose-finding study: a 12-week course of treatment with 5, 8, 10, or 13 g daily of the fungal extract in postmenopausal women previously diagnosed with breast cancer but no longer undergoing treatment and who are recurrence free. Aromatase inhibition by aromatase activity (AA) tests and a decrease in free estradiol (FE2) and cytokine levels were the counted parameters. No subject met the predefined response criterion, but despite this, it was observed that FE2 tended to increase over the 12-week treatment period for the 5 g and 8 g dose groups, while it remained stable for the 10 g and 13 g groups; in the first two groups, AA testing also revealed a substantial increase in post-prandial peak, which decreased in the 10 g group and disappeared in the 13 g group. In addition, albeit without a clear dose–effect correlation, there were increases in IL-1β and IL-2, and a decrease in IL-6. The authors concluded that antiaromatase bioactive compounds were present in plasma at a consumption level of 10–13 g of extracts (corresponding to 100–130 g of the whole mushroom), but not in sufficient concentration to cause a significant reduction in estrogen, at least over the time period evaluated.

A different approach was used to evaluate another aspect of WBM, namely as a food rich in prebiotics. Health effects and the gut microbiota were assessed in an open-label crossover trial by subjecting 32 healthy adults to the consumption of protein-matched amounts of mushrooms or meat twice daily for ten days [115]. No differences were found between the two groups in breath hydrogen, stool frequency, consistency, fecal pH, or Short Chain Fatty Acid (SCFA) concentrations.

On the other hand, the composition of the fecal microbiota of WBM-fed subjects was different, with a higher abundance of Bacteroidetes and a lower abundance of Firmicutes. Besides, the impact of mushroom consumption on laxation and consequently on gut health can be deduced from the increase in stool weight and the presence of undigested mushrooms in the stool.

Still dealing with oyster mushroom, an interesting study by Jesenak et al. [116] highlights the efficacy of pleuran in preventing morbidity in children due to recurrent respiratory tract infections (RRTIs), while also outlining its complex immunomodulatory activity. In this double-blind, placebo-controlled, randomized, multicenter trial, 175 children with more than five respiratory infections were randomized to receive 5 mL/5 kg Imunoglukan P4H^®^ (Nové Záhrady I č., Bratislava, Slovak Republic) syrup (10 mg pleuran and 10 mg vitamin C in 1 mL of syrup) or placebo (only vitamin C, 10 mg/mL) for 6 months. Blood samples and questionnaires were taken for 12 months. A higher proportion of the treated group compared to the placebo group did not suffer from any respiratory infection throughout the treatment. Furthermore, in the P4H group, there were significant reductions in the frequency of flu and flu-like disease and the number of lower respiratory tract infections, as well as a statistically significant modulation of humoral and cellular immunity. In particular, in the treated group, the concentration of IgG and IgM increased during the treatment period and remained heightened throughout the study; IgA increased more in the P4H group than in the placebo one; Immunoglukan was also associated with an increase in NK cells and prevented the decline in CD8+ T cytotoxic lymphocytes.

Pleuran has also been tested for the management of herpes simplex virus type I infection, as the typical remedy acyclovir can cause viral resistance if used for long periods. Urbancikova et al. [117] conducted a randomized, placebo-controlled clinical trial to assess the effect of this compound on the duration and intensity of herpes symptoms and the incidence rate and duration of acute respiratory symptoms and intercurrent diseases in HSV-1-positive patients. This was done in 90 patients randomly assigned to receive pleuran or placebo, and the study was divided into two phases: an acute treatment phase, lasting 10 days, in which either Immunoglukan P4H^®^ ACUTE (300 mg pleuran, 160 mg vitamin C, 10 mg Zn) or placebo (160 mg vitamin C, 10 mg Zn) was administered daily; and a subsequent 120-day preventive phase, with daily administration of either Immunoglukan P4H^®^ (100 mg pleuran, 100 mg vitamin C) or placebo (100 mg vitamin C). Active treatment resulted in a significant shortening of the duration of herpes symptoms. During the preventive phase, the duration and severity of respiratory symptoms were lower. In both phases, no side effects were observed.

## 5. Discussion

Medicinal mushrooms have been shown to have many different pharmacological properties and are the subject of increasing interest. Many of them are already being used, particularly in the field of oncology, for their immunomodulatory and antitumor actions, which complement traditional treatments, improving their action and reducing their side effects [1,3,5].

The medicinal properties of mushrooms are due to the numerous and diverse secondary compounds and metabolites present in the mycelial and/or carpophore structures, which can act, in a synergistic or non-synergistic manner, on various biological functions of the human organism. All these properties were initially detected, demonstrated, and elucidated by in vitro tests, the first necessary step in highlighting the relevant potential of a given fungus for therapeutic purposes, followed by in vivo tests on animal models, usually murine, for the first investigations of the effects of the substances in living cells and systems. Nevertheless, although this research aims to develop drugs and natural products for human health care, few studies have been conducted within the framework of clinical trials, a domain that is still rather unstructured, with numerous shortcomings and gaps. As there is no standard for the procedure and evaluation of results, the value of such studies is undermined in several respects. First of all, the basic experimental design, which often examines a sample that is too small and therefore not sufficiently representative, and can lead to false positive results [103,118]. Moreover, not all trials are randomized or have a placebo control [104,105], nor are double-blinded, and there is generally a lack of uniqueness of purpose, so only some parameters are taken into account and others are neglected, such as safety and side effects. Several, then, are not double-blinded, so the variable of uncontrollable human bias comes into play. Some studies also rely on the methodology of subjective assessments of certain parameters, such as quality of life, so the result examined may not have any real scientific value [66,69,87].

Again, in most cases, the so-called pilot or phase I studies, which are normally designed to assess the efficacy of a preparation, identify its dosage range, and test its safety, and thus see whether a given research project can be continued, have no follow-up. The results thus remain only preliminary, far from a medical application procedure.

All these factors also make it difficult to compare the results obtained in separate clinical studies. In this regard, it must be emphasized that another factor also comes into play, namely the preparation used to carry out the trial. Even if the same fungal species is tested, sometimes opposite or different results are obtained, as in the case of Yoshimura et al. [68] and Ohno et al. [69] with *A*. *blazei* Murrill. Alternatively, a fungus tested for its specific action, which has already been proven in other trials, turns out to be completely ineffective, if not actually deleterious, worsening the clinical picture [77,78]. This is also explained by the type of extract used, either because the extraction method influences the substances present or their activity, or because the concentration of a given metabolite in the extract cannot be ascertained. Furthermore, even under the same conditions, the medicinal properties of a given mushroom can vary enormously depending on the strain, the geographical area, the growing conditions and substrate used, the part of the mushroom used, and the growing stage at the moment of processing. All these parameters change the composition of the mushroom and, consequently, its bioactive capacity.

## 6. Concluding Remarks

In recent years, research into medicinal mushrooms has progressed exponentially, but much remains to be done. Many species remain unstudied or underestimated in terms of their pharmacological properties. The primary urgency is also to identify the molecules present in the extracts, the metabolites responsible for their effects, their chemical characterization, and their mechanism of action. There is also an urgent need to fully understand both their individual and synergistic actions, with particular attention paid to in vivo dynamics, as well as refining the design of in vivo and clinical studies. It is also necessary to standardize the production of mushroom supplements throughout the supply chain, from cultivation to the extraction and preparation of the commercial formulation, as well as precise monitoring and regulation to ensure high quality levels.

## Abbreviations

ABM	*Agaricus blazei* mushroom
ACC	Acetyl-CoA Carboxylase
ALT	Alanine Aminotransferase
AOX	Alternative Oxidase
ASCT	Autologous Stem Cell Transplantation
AST	Aspartate Aminotransferase
ATA	Anctin-A
BG	Antibody Response to β-glucan
BRMs	Biological Response Modifiers
CAM	Complementary Alternative Medicine
cAMP	Cyclic Adenosine Monophosphate
CDC	Complement-Dependent Cytotoxicity
CIM	Complementary Integrated Medicine
CLA	Conjugated Linoleic Acid
ConA	Concanavalin A
DBP	Diastolic Blood Pressure
DCs	Dendritic Cells
DC-STAMP	Dendritic Cell-Specific Transmembrane Protein
ER	Endoplasmic Reticulum
EFSA	European Food Safety Authority
EORTC-QLQ-C30	European Organisation Research Treatment Cancer Core Quality of Life Questionnaire C30
FAC	Fanconi Anemia (FA) group C gene
FACTF	Functional Assessment of Cancer Therapy Fatigue
FAS	Fatty Acid Synthase
FDA	American Food and Drug Administration
FSG	Fasting Serum Glucose
FIPs	Fungal Immunomodulatory Proteins
FSI	Fasting Serum Insulin
GFP	*Grifola frondosa* Polysaccharide
hAMSCs	Human Amniotic Mesenchymal Cells
HADS	The Hospital Anxiety and Depression Scale
HDL	High-Density Lipoprotein
HDL-C	High-Density Lipoprotein Cholesterol
HLA	Human Leukocyte Antigens
HOMA-IR	Homeostasis Model Assessment of Insulin Resistance
iROS	Intracellular Reactive Oxygen Species
IgG	Immunoglobulin
IL	Interleukin
LDL	Low Density Lipoproteins
LMW	Low-Molecular-Weight
MPP	1-methyl-4-phenylpyridinium
NOS	Nitric Oxide Synthase
IR	Insulin Receptor
IRS-1	Insulin Receptor Substrate-1
LPS	Lipopolysaccharide
MAPK	Mitogen-Activated Protein Kinase
MITF	Microphthalmia-Associated Transcription Factor
MMs	Medicinal Mushrooms
MMP-2	Matrix Metalloproteinase-2
NK	Natural Killer
NP	Nanoparticle
PAMP	Pathogen-Associated Molecular Pattern
PBMCs	Peripheral Blood Mononuclear Cells
PD	Parkinson disease
PHA	Phytohemagglutinin
PSA	Prostate-Specific Antigen
PSK	*Polysaccharide*-K (Krestin)
PSP	Polysaccharide Peptide
RCT	Randomized Clinical Trial
SBP	Systolic Blood Pressure
SCFA	Short Chain Fatty Acid
SREBP-1c	Sterol Regulatory Element-Binding Protein 1
TAC	Transverse Aortic Constriction
TGs	Plasma Triglycerides
TNBC	Triple-Negative Breast Cancer
TNF-α	Tumor Necrosis Factor-α
TRP-1	Tyrosinase-Related Protein-1
VEGF	Vascular Endothelial Growth Factor
